# Use of magnetic resonance imaging for diagnosis and after treatment of patients with myeloid sarcoma of the brain

**DOI:** 10.18632/oncotarget.21905

**Published:** 2017-10-13

**Authors:** Xuewen Hou, Longting Du, Haitao Yu, Xiaojin Zhang

**Affiliations:** ^1^ Department of Radiology, Baoan Central Hospital of Shenzhen, Baoan District, Shenzhen 518102, China; ^2^ Department of Radiology, Aerospace Center Hospital, Haidian District, Beijing 100049, China

**Keywords:** brain myeloid sarcoma, magnetic resonance imaging, diffusion weighted imaging, arterial spin labeling imaging, susceptibility weighted imaging

## Abstract

The purpose of this retrospective study was to assess the utility of magnetic resonance imaging (MRI) for evaluating post-treatment responses in patients with myeloid sarcoma (MS) of the brain. We evaluated images from both conventional and advanced MR, including diffusion weighted imaging (DWI), arterial spin labeling (ASL) and susceptibility weighted imaging (SWI). Parameters of our qualitative review included lesion location, number, size, morphologic characteristics, surrounding edema, mass effect, pattern and degree of enhancement, ± restricted diffusion, ±susceptibility artifact and ± higher perfusion. Our quantitative assessments were calculated from DW and ASL MR images. The 10 patients had a total of 40 lesions in their brains (mean lesion size of 2.0 ± 0.8 cm). The majority of cases exhibited restricted diffusion (90%) and mild-to-moderate low perfusion (80%). Follow-up MRI after chemotherapy revealed that most lesions (80%) were significantly alleviated after two chemotherapy courses and further improved after four courses. Only a few lesions (5%) were residual after six courses. These findings demonstrate that brain MS can be characterized by changes in various MRI parameters and that MRI can be a useful and predictive assessment tool for brain MS diagnosis and treatment management.

## INTRODUCTION

Myeloid sarcoma (MS) is an extramedullary tumoral mass composed of immature myeloid cells. MS can manifest from various clinical conditions, including acute myeloid leukemia (AML), myeloproliferative disorders, myelodysplastic syndromes (MDS) and isolated tumors in patients without evident hematological disorders [1, 2]. In 2008 the World Health Organization classification adopted the term “myeloid sarcomas” as a subgroup of “acute myeloid leukemias, not otherwise categorized” [3]. The most frequently involved sites of MS are soft tissue, bone, skin and lymph node [4], while rarely involving the central nervous system [5]. Clinical symptoms of MS depend on the organs involved and generally do not impact treatment response [6]. Early diagnosis of MS and prompt treatment is associated with improved survival [7].

There are several case reports [8, 9] and pictorial essays [10] in radiology literature that report on image performance in MS, however, there exists only 5 small studies that systematically analyzed MS [11–15]. Choi et al. [11] previously reported computed tomography (CT) findings of MS involving the bowel, and Noh et al. [12] first reported CT findings of MS involving the head and neck. Seok et al. [13] reported MRI (pre-contrast T1WI and post-contrast T2WI) findings of MS involving the spine. In addition, Shinagare et al. [14] systematically reviewed and described multisystem CT and MRI (T1WI, T2WI and T1 post-contrast) characteristics of MS, but only 6 patients with central nervous system involvement were studied. Moreover, Chaudhry et al. [15] described CT and MRI (T1WI, T2WI and contrast-enhanced T1WI, DWI and SWI) characteristics of MS involving the brain. None of these studies systematically performed qualitative and quantitative analysis of diffusion and perfusion characteristics of MS, and none of these studies assessed treatment response of MS using MRI. The aim of this study was to qualitatively assess MRI findings of brain MS, perform quantitative analysis and to assess MRI as a tool to evaluate treatment response of brain MS.

## MATERIALS AND METHODS

### Study group

We performed a computer search of the pathology, radiology and medical records of 2 tertiary care centers in Beijing and Shenzhen, China (Baoan Central Hospital of ShenZhen and Aerospace Central Hospital) to identify pathologically confirmed cases of myeloid sarcoma that underwent treatment with standard chemotherapy from January 2011 to December 2016. We identified 42 patients with pathologically confirmed myeloid sarcoma anywhere in the body, 18 patients with myeloid sarcoma involving the brain, but only 10 patients (6 male and 4 female; range, 10–45 years) that underwent MRI examination and chemotherapy. These 10 patients constituted our study cohort.

### Imaging protocol

The sequence of our brain tumor imaging protocol was as follows: T2-weighted imaging, fluid-attenuated inversion recovery imaging, non-enhanced T1-weighted imaging, Gd-DTPA contrast-enhanced T1-weighted imaging, SW imaging, DW imaging and ASL perfusion imaging. Images were from MRI examinations that utilized GE 1.5T and Siemens 3T MR imaging systems. Follow-up MRI was performed at 2 chemotherapy courses, 4 chemotherapy courses and 6 chemotherapy courses after treatment.

### Qualitative image analysis

Two radiologists independently reviewed and evaluated the MR images with regard to the locations, morphologic characteristics, architecture, degree of surrounding edema and mass effect, pattern and degree of enhancement, ± susceptibility artifact, ± restricted diffusion and ± higher perfusion. The MRI signal intensity of the lesions was compared to the contralateral normal gray matter. The degree of enhancement, surrounding edema and mass effect were subjectively assessed as mild, moderate or marked.

### Quantitative DW Image and ADC value analysis of brain MS

MRI images were transferred to the MR protocol workstation. From conventional MR and DW images, 2 independent and blinded observers identified the tumors’ contrast-enhanced components. The observers were allowed to adjust the grayscale center level and window width settings, as well as the zoom factor for optimal image interpretation. They measured ADC values of tumor by manually placing regions of interest (ROIs) within tumor components on ADC maps. ROIs (40–60 mm^2^) were placed to avoid cystic and/or necrotic areas. Each reviewer placed 3–5 ROIs on areas viewed to be the minimum and maximum ADC regions. There were more ROIs for more heterogeneous lesions. The average ADC was calculated as the mean of the summed values of the 3–5 ROIs. Minimum and maximum ADCs were selected, and the same observer placed ROIs (approximately 2.5 cm^2^/ROI) in contralateral normal hemispheres. The resulting ADC parameter measurements computed for each lesion were averaged between the 2 observers. Mean ADC values between 2 locations were compared with the independent samples *t*-test. *P* values < 0.05 were considered statistically significant. Statistical software (SPSS, version 22.0) was used to perform statistical analyses.

### Quantitative ASL perfusion and CBF value analysis of brain MS

ASL perfusion parametric maps were obtained by using a dedicated software package and other software (Matlab 2013b; Mathworks). In the same way, tumor regions of interest (ROIs) were determined and manually drawn in an area with maximum signal enhancement on grayscale, and ASL cerebral blood flow (CBF) maps were calculated using perfusion software by 2 independent observers. Necrotic tissue and large vessels were avoided with T2-weighted images, CE T1-weighted images and ASL images. Tumor Blood Flow (TBF) values were measured in the ROIs described above. Each reviewer placed 3–5 ROIs on areas viewed to be the minimum and maximum TBF regions. The mean TBF values were calculated by taking the mean of the summed values of the 3–5 ROIs. Minimum and maximum CBF values were selected, and ROIs were also placed by the same observers in contralateral normal hemispheres. Hemisphere ROIs (approximately 2.5 cm^2^/ROI) were placed centrally within the hemispheres. The resulting CBF parameter measurements computed for each lesion were averaged between the 2 observers. The mean CBF values between the two locations were compared with independent samples *t*-test. *P* values < 0.05 were considered statistically significant. Statistical software (SPSS, version 22.0) was used to perform statistical analyses.

### Evaluation of post-treatment response of brain MS

All patients underwent standard induction chemotherapy and intrathecal chemotherapy, and 4 patients also underwent radiation treatment. Standard chemotherapy treatment of MS is identical to that of AML, comprised of 2 courses of induction chemotherapy (idarubicin and arabinocytidine) and 4 courses of consolidation chemotherapy. Treatment response is classified as complete remission, partial remission or recrudescence. To assess MS treatment response via MRI, the loss of mass suggests a complete remission of MS. Although, the mass does not completely disappear, but shrinks, suggesting partial remission. If the tumor persists, it indicates residual disease. Radiologists independently reviewed and evaluated the post-treatment MR images with regards to changes in lesion size, surrounding edema, mass effect, degree of enhancement, signal intensity change of DWI images and post-treatment MRI.

## RESULTS

### Clinical findings

As shown in Table 1, the most common clinical symptom of brain MS was headache (8 of 9 patients). Other manifestations included numbness of limb, blurred vision and epilepsy. All patients have a known history of hematologic malignancy, including 7 patients with a known history of AML, 2 patients with a history of acute lymphoblastic leukemia (ALL) and 1 patient with a history of chronic myelogenous leukemia (CML). Primary myeloid sarcoma was not found in our study.

**Table 1:** Clinical characteristics of patients with intracranial myeloid sarcoma

Patient No.	Sex	Age	Disease and Diagnosis Time	Clinical Manifestation at Presentation
1	M	20	AML/6 months	Numbness of limb
2	M	39	CML/120 months	Headache
3	M	25	ALL/11 months	Headache, nausea and vomiting
4	M	38	AML/2 month	Headache and blurred vision
5	F	10	AML/24 months	Headache
6	F	29	AML/21 months	Headache
7	M	23	AML/12 months	Epilepsy and headache
8	M	26	ALL/24 months	Headache, nausea and vomiting
9	F	45	AML/8 months	Headache
10	F	31	AML/16 months	Headache

### Qualitative MRI of brain MS

**Figure 1:**
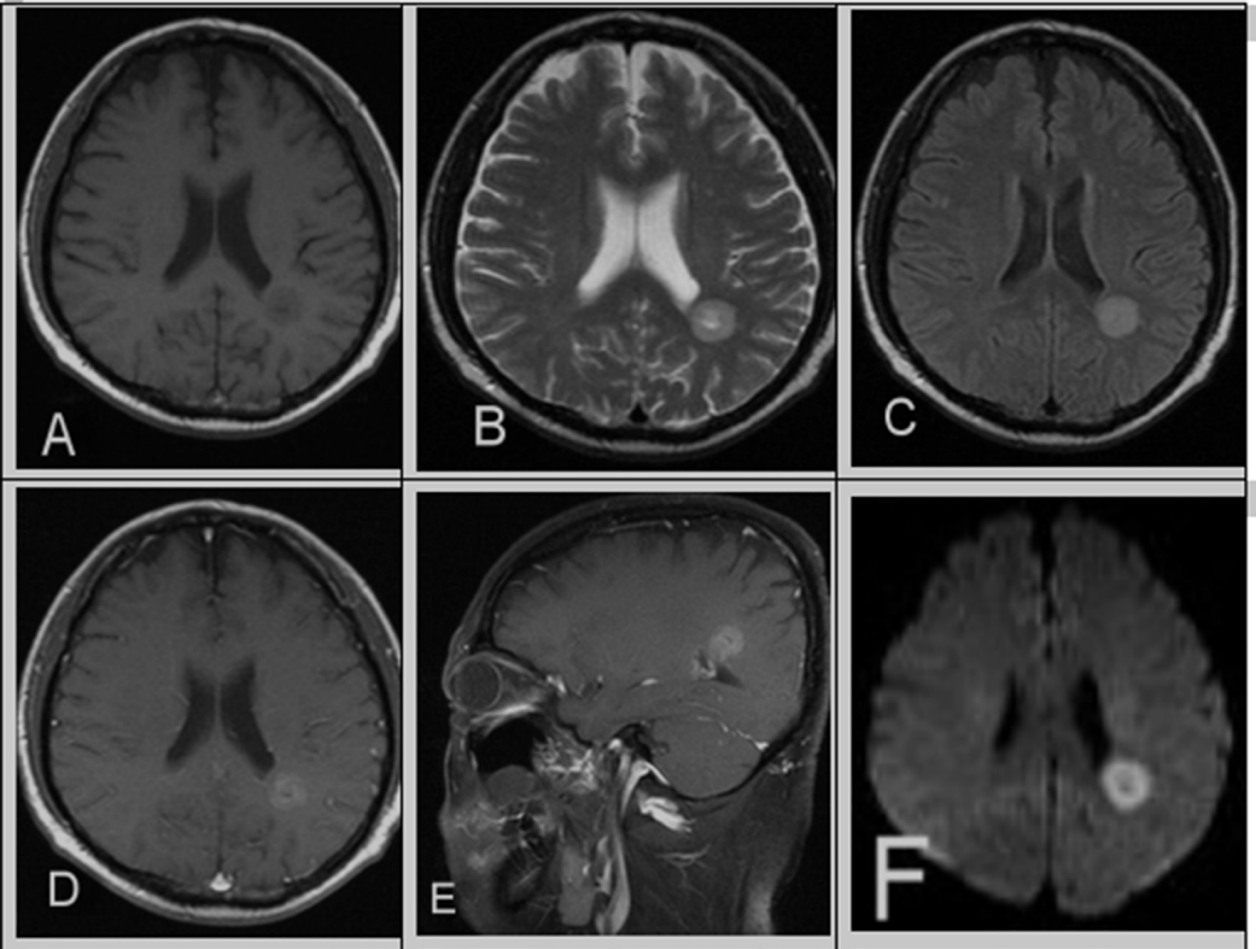
A 39-year-old male with a 10-year history of CML in relapse presents with headaches (**A**) Unenhanced T1WI revealed a well-defined, heterogeneous brain parenchyma mass with mild hypointensity. **(B)** T2WI and **(C)** FLAIR showed hyperintensity, post-contrast. **(D, E)** T1WI showed mild hyperintensity and cystic changes. **(F)** DWI showed evidence of restricted diffusion in the corresponding area. There was no surrounding vasogenic edema or mass effect.

**Figure 2:**
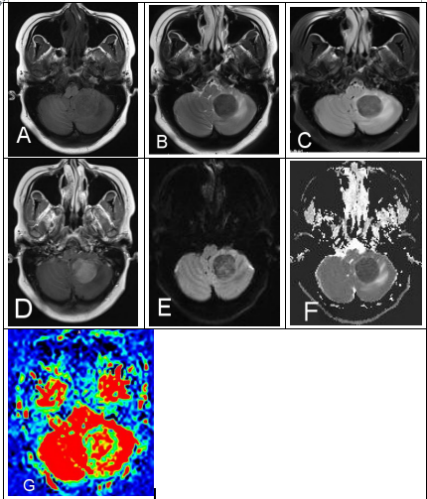
A 29-year-old female with a history of AML in remission presents with headaches, nausea and vomiting. (A) MRI demonstrated left cerebellum mass unenhanced T1WI, **(B)** T2WI, **(C)** FLAIR isointense-to-mild hypointense and **(D)** CE-T1WI showed homogenous hyperintensity. **(E)** DWI and **(F)** ADC map demonstrated mild-to-moderate restricted diffusion. **(G)** ASL showed mild hypointensity with mild surrounding vasogenic edema and mass effect.

Qualitative results of brain MS are shown in Table 2. A total of 40 lesions in the brain were studied, including lesions located in the brain parenchyma involving the temporal lobe (*n* = 10), frontal lobe (*n* = 15), parietal lobe (*n* = 6), occipital lobe (*n* = 3) and cerebellar hemisphere (*n* = 6). Multifocal brain lesions were noted in 6 patients. On MRI, mean lesion size was recorded as 2.0 ± 0.8 cm. The lesions were isointense (70%) or hypointense (30%) on T1-weighted images with homogeneous (75%) or heterogeneous (25%) enhancement. On fluid-attenuated inversion recovery and T2-weighted images, lesions were hyperintense (90%) or isointense (10%), with surrounding vasogenic edema (75%) or obvious mass effect (10%). For those with surrounding edema and mass effect, about 50% had mild edema, 15% had mass effect and 10% had cysts and/or necrosis (Figure 1). On DWI, the lesions (90%) demonstrated restricted diffusion. On SWI or T2*-GRE imaging, lesions (12.5%) revealed susceptibility artifact. None of these had evidence of observable calcification. On ASL perfusion imaging, the lesions (80%) demonstrated mild-to-moderate lower-perfusion (Figure 2). Two patients had diffuse infiltration of the multifocal meninges. Moreover, 1 patient had involvement of the skull.

**Table 2:** Qualitative MRI findings in myeloid sarcoma

Total no. of lesions	40			
**Location**	temporal lobe	10	25%	10/40
	frontal lobe	15	37.5%	15/40
	parietal lobe	6	15%	6/40
	occipital lobe	3	7.5%	3/40
	Cerebellar hemisphere	6	15%	6/40
**Mean lesion size**	2.0 ± 0.8 cm			
**Shape**	regular	36	90%	36/40
	irregular	4	10%	4/40
**Signal intensity**				
**T1WI**	isointense	28	70%	28/40
	Hypointense	12	30%	12/40
**T2WI/FLAIR**	Hyperintense	34	85%	34/40
	Isointense	4	10%	4/40
	Hypointense	2	5%	2/40
**SWI**	Isointense	35	87.5%	35/40
	Hypointense	5	12.5%	5/40
**DWI**	Hyperintense	32	80%	36/40
	Isointense	4	10%	4/40
**ASL**	Isointense	6	15%	12/40
	Mild Hypointense	26	65%	20/40
**Degree of contrast enhancement**	Mild	6	15%	6/40
	Moderate	12	30%	12/40
	Marked	4	10%	4/40
**Homogeneity of Contrast enhancement**	Homogeneous	30	75%	30/40
	Heterogeneous	10	25%	10/40
**Degree of surrounding edema**	Mild	20	50%	12/40
	Moderate	8	20%	8/40
	Marked	2	5%	2/40
**Mass effect**	Mild	4	10%	4/40
	Marked	2	5%	2/40
**Cysts and/or necrosis**		4	10%	2/40
**Calcification**		0	0%	0/40

### Quantitative ADC values of brain MS

Maximum ADC, mean ADC, minimum ADC and *P* values are shown in Table 3. The maximum ADC value of brain MS (450 ± 60 × 10^-6^ mm^2^/sec) is lower than the minimum ADC value of normal brain (600 ± 60 × 10^-6^ mm^2^/sec). The mean and minimum ADC values of brain MS are lower than the ADC values of normal brain. This shows a marked restricted diffusion in brain MS. *P* = 0.001.

**Table 3:** ADC Values in myeloid sarcoma and normal brain

ADC of MS	Maximum ADC	450 ± 60 ×10^−6^ mm^2^/sec
	Minimum ADC	240 ± 40 ×10^−6^ mm^2^/sec
	Mean ADC	400 ± 55 ×10^−6^ mm^2^/sec
ADC of Normal Hemisphere	Maximum ADC	750 ± 110 ×10^−6^ mm^2^/sec
	Minimum ADC	600 ± 70 ×10^−6^ mm^2^/sec
	Mean ADC	660 ± 70 ×10^−6^ mm^2^/sec
*P* value	*P* = .001	

### Quantitative CBF values of brain MS

TBF and *P* values are shown in Table 4. The maximum TBF value of brain MS (46.06 ± 10.80 ml/min.100 g) is lower than the minimum CBF value of normal brain (52.08 ± 10.36 ml/min.100 g). The mean and minimum TBF values of brain MS were also lower than the CBF values of normal brain. This shows a mild-to-moderate lower perfusion in brain MS. *P* = 0.01.

**Table 4:** TBF and CBF values in myeloid sarcoma and normal brain

**TBF of MS**	Maximum TBF	46.06 ± 10.80 ml/(min.100 g)
	Minimum TBF	28.66 ± 10.22 ml/(min.100 g)
	Mean TBF	40.56 ± 11.32 ml/(min.100 g)
**CBF of Normal Hemisphere**	Maximum CBF	65.55 ± 11.20 ml/(min.100 g)
	Minimum CBF	52.08 ± 10.36 ml/(min.100 g)
	Mean CBF	60.18 ± 12.64 ml/(min.100 g)
***P*** **value**	*P* = .01	

### Evaluation of post-treatment response of brain MS

**Figure 3:**
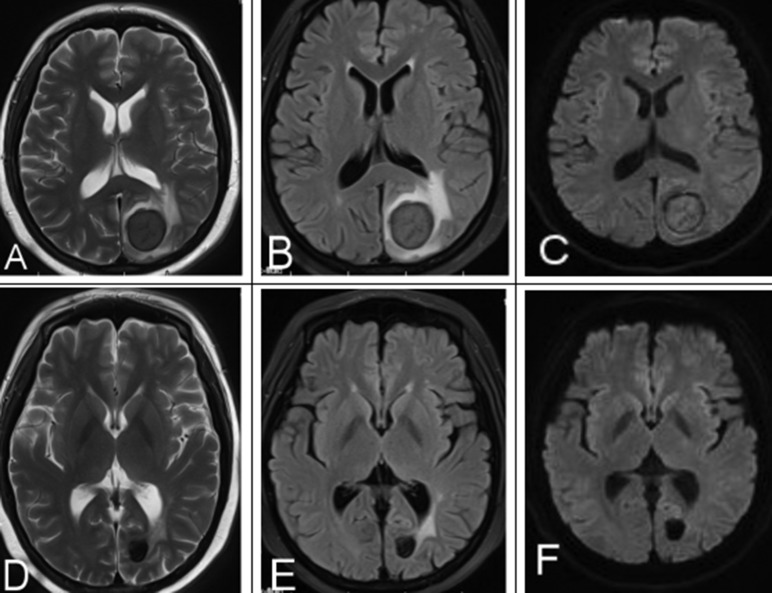
A 25-year-old female with a history of AML underwent chemotherapy and MRI follow-up (**A–C**) MRI signal intensity was obvious and lower than pre-treatment on T2WI, FLAIR, DWI, indicating a good treatment response. (**D–F**) Post-treatment mass on T2WI, FLAIR, DWI.

All patients underwent standard induction chemotherapy and intrathecal chemotherapy, and 4 patients also underwent radiation treatment. MRI follow-up was performed at 2 chemotherapy courses, 4 chemotherapy courses, 6 chemotherapy courses and after treatment. 80% of lesions achieved complete remission. MRI showed that these lesions turned into a cavity and were characterized by obvious hypointensity on T2WI/FLAIR and DWI versus pretreatment (Figure 3). 15% of lesions were further improved after 4 chemotherapy courses, as indicated by gradually lower MR signal intensities. 5% of lesions indicated residual disease after 6 chemotherapy courses, as indicated by hyperintensity on DWI, ADC map and CE-T1WI.

## DISCUSSION

Myeloid sarcoma (MS) is a rare manifestation of leukemias, myeloproliferative neoplasias or myelodysplastic syndromes and is composed of immature myelomonocytic cells in extramedullary sites. MS can involve any organ, but the most common sites include soft tissue, bone, skin and lymph node [4]. Survival outcomes are markedly improved when patients are diagnosed and treated early with standard induction chemotherapy or radiation [16].

Myeloid sarcoma of the central nervous system (CNS) is a rare presentation of leukemic mass infiltration outside of the bone marrow involving extramedullary sites. On rare occasions, it can also invade the brain parenchyma. Notably, all 40 lesions in our study were located in the brain parenchyma.

There are a few studies that have previously systematically analyzed CNS MS. Shinagare et al. [14] and Chaudhry et al. [15] both described brain MS lesions showing isointensity on T1WI, hyperintensity on T2WI and hyperintensity on FLAIR sequences. Similar to our study, their qualitative assessment of lesions demonstrated a restricted diffusion on DWI and revealed few susceptibility artifacts on GRE. Qualitative assessment of MS on ASL is unique to our study and demonstrated mild-to-moderate low perfusion. In a case report [17], Hakyemez et al. reported that lesions were low perfusion on dynamic susceptibility contrast (DSC) perfusion-weighted imaging (PWI) of intracranial myeloid sarcoma. We can consider these results as similar to our study. On the other hand, our study was unique in it exploration of surrounding edema and mass effect. Lesions were predominantly mild surrounding edema, and there was little mass effect. We also found cysts and/or necrosis in very few MS lesions (10%) on T2WI, FLAIR and CE-T1WI. Notably, none of the lesions had any observable calcium.

To our understanding, usage of DWI to quantitatively assess brain MS has not yet been reported. The underlying rationale of DWI is based upon the fact that narrowed extracellular space decreases the diffusion of water molecules in the extracellular space. Restricted diffusion is quantitatively measured by the apparent diffusion coefficient (ADC), which is lower in highly cellular tumors and higher in cases of edema and necrosis. DW imaging has been extensively investigated as an important diagnostic tool for the assessment of primary brain tumors, and tumor grade has been confirmed to correlate with the ADC values. High-grade brain tumors have lower ADC values than low-grade tumors and normal appearing parenchyma [18, 19]. In fact, some studies have shown that ADC value can grade for gliomas [20, 21]. In accordance with other malignant brain tumor studies, our ADC measurements of brain MS revealed mild-to-moderate values (*P* = 0.001 (less than .05). Based on these results, we consider the cases we studied to have mild-to-high cell density, but inferior-to-high-grade brain tumors. These results are consistent with MS pathological observations and are supportive of the prospect of utilizing brain MS ADC values to identify patients with high-grade brain tumors.

Furthermore, ADC values are useful in assessing treatment responses of brain tumors. For example, an increase in ADC after treatment is considered a good response, but a decrease in ADC after treatment is associated with tumor progression [22, 23].

To our understanding, usage of ASL to quantitatively assess brain MS has not yet been reported. ASL is an MRI perfusion technique that can be used to assess brain tumor vascularity and offer absolute quantification of CBF noninvasively. Meningiomas have high vascularity and showed the highest TBF values [24], and high-grade gliomas have higher TBF values than low-grade gliomas [25]. CNS lymphomas were found to have a mean TBF [26]. In our study, measurement of TBF revealed mild-to-moderate low TBF values in brain MS (P = 0.01), which is indicative of low perfusion in brain MS, similar to CNS lymphomas. We think it is beneficial to differentiate brain MS and other high-vascularity brain tumors, such as high-grade gliomas and meningiomas, but it is difficult to differentiate between brain MS and CNS lymphomas on ASL perfusion images.

A brain mass in a patient with a history of hematologic conditions, especially an AML, should be considered MS. Characteristic of brain MS, the MRI findings in our study were homogeneous non-calcified masses, which are T1 isointense and T2/FLAIR hyperintense with homogeneous enhancement, having mild surrounding vasogenic edema, no obvious mass effect, few cysts and/or necrosis, occasional susceptibility artifact, restricted diffusion and mild-to-moderate low ADC values, low perfusion and mild-to-moderate low TBF values. These findings have certain imaging characteristics. Our study was designed to narrow differential diagnoses by excluding inflammatory, hemorrhagic and calcific lesions. In addition, quantitative assessment of ADC and CBF can help differentiate some other brain tumors. Notably, in the diagnosis of brain MS, it is important to utilize both MRI findings and cerebrospinal fluid analysis.

MRI is a very useful method for assessing MS treatment response. Follow-up MRI found that most MS lesions turn into a cavity characterized by obvious hypointensity on T2WI/FLAIR, DWI and CE-T1WI as compared with pre-treatment. We consider these brain MS cases to have achieved complete remission. Few lesions were still hyperintense on T2WI/FLAIR, DWI, and CE-T1WI after treatment with 6 chemotherapy courses. This is indicative of residual disease, which was confirmed by cerebrospinal fluid analysis.

Our study did have some limitations. Firstly, we only included a small number of patients due to the rarity of cases with tumor in the brain parenchyma. Secondly, pathological confirmation was no acquired for every mass lesion observed in the brain. However, we believe that at least 1 pathologically proven MS in the brain in each patient provided sufficiently compelling evidence. Thirdly, we were unable to obtain a primary brain MS patient in this study due to the rarity of this tumor type. Finally, our study did not include SWI semi-quantitative assessment of intratumoral susceptibility signals (ITSS) of the brain MS lesions.

MS of the brain is a rare manifestation of extramedullary leukemia that is mostly present in AML patients. MS of the brain may be characterized by changes in MRI intensity, contrast enhancement, diffusion, perfusion and susceptibility artifact, which is highly predictive in patients with a history of hematologic disorder. MRI can become a useful assessment tool for diagnosis of MS and treatment management.

## Abbreviations

MS: myeloid sarcoma
DWI: diffusion weighted imaging
ASL: arterial spin labeling imaging
SWI: susceptibility weighted imaging
